# Harnessing adrenergic blockade in stress-promoted TNBC *in vitro* and solid tumor *in vivo*: disrupting HIF-1α and GSK-3β/β-catenin driven resistance to doxorubicin

**DOI:** 10.3389/fphar.2024.1362675

**Published:** 2024-06-19

**Authors:** Yasmeen Attia, Andrew Hakeem, Rawda Samir, Aya Mohammed, Abdullrahman Elsayed, Alaa Khallaf, Eman Essam, Hossameldeen Amin, Sarah Abdullah, Salwan Hikmat, Tarek Hossam, Ziad Mohamed, Ziad Aboelmagd, Olfat Hammam

**Affiliations:** ^1^ Pharmacology Department, Faculty of Pharmacy, The British University in Egypt, El-Sherouk City, Egypt; ^2^ Health Research Center of Excellence, Drug Research and Development Group, Faculty of Pharmacy, The British University in Egypt, El-Sherouk City, Egypt; ^3^ Faculty of Pharmacy, The British University in Egypt, El-Sherouk City, Egypt; ^4^ Pathology Department, Theodor Bilharz Research Institute, Giza, Egypt

**Keywords:** β-catenin, cancer stem cells, carvedilol, doxorubicin, GSK-3β, HIF-1α, stress, triple negative breast cancer

## Abstract

Sympathetic activation triggered by chronic stress afflicting cancer survivors is an emerging modulator of tumorigenesis. Adrenergic blockade was previously associated with improving response to doxorubicin (DOX) in triple-negative breast cancer (TNBC), yet the precise underlying mechanisms remain obscure. The resilience of cancer stem cells (CSCs) during chemotherapy fosters resistance and relapse. Hypoxia-inducible factor-1α (HIF-1α) and β-catenin are intertwined transcriptional factors that enrich CSCs and evidence suggests that their expression could be modulated by systemic adrenergic signals. Herein, we aimed to explore the impact of adrenoreceptor blockade using carvedilol (CAR) on DOX and its potential to modulate CSCs overcoming chemoresistance. To achieve this aim, *in vitro* studies were conducted using adrenaline-preincubated MDA-MB-231 cells and *in vivo* studies using a chronic restraint stress-promoted solid tumor mouse model. Results revealed that adrenaline increased TNBC proliferation and induced a phenotypic switch reminiscent of CSCs, as evidenced by enhanced mammosphere formation. These results paralleled an increase in aldehyde dehydrogenase-1 (ALDH-1) and Nanog expression levels as well as HIF-1α and β-catenin upsurge. *In vivo*, larger tumor volumes were observed in mice under chronic stress compared to their unstressed counterparts. Adrenergic blockade using CAR, however, enhanced the impact DOX had on halting TNBC cell proliferation and tumor growth via enhanced apoptosis. CAR also curbed HIF-1α and β-catenin tumor levels subsequently suppressing ALDH-1 and SOX2. Our study unveils a central role for HIF-1α linking stress-induced sympathetic activation fueling CSC enrichment via the β-catenin pathway. It also highlights novel insights into CAR’s capacity in reversing DOX chemoresistance in TNBC.

## 1 Introduction

Triple-negative breast cancer (TNBC) is a coalition of diverse breast cancers only unified by the common feature of lacking expression of hormone receptors (estrogen and progesterone) and human epidermal growth factor receptor-2 (HER2) (Marra et al., 2020). This lack of actionable targets lays discourse for conventional chemotherapies to lead the limited treatment options clinically adopted for TNBC patients (Bianchini et al., 2016). While these systemic therapeutic modalities effectively kill the bulk of TNBC tumors by targeting rapidly proliferating cells, a subset of dormant stem-like cells survive and reconstitute tumors upon treatment cessation. Thus, these cells, dubbed cancer stem cells (CSCs), are often targeted in TNBC and other cancers to subdue recurrence and chemoresistance (Zhou et al., 2021).

The tumor microenvironment (TME) constitutes a dynamic landscape that is incessantly remodeled by a vast set of cues derived locally as well as systemically from alterations experienced by the afflicted patient (Jin and Jin, 2020). One key feature of the solid TME is its hostile hypoxic niche to which cancer cells adapt by inducing the expression of hypoxia inducible factors (HIFs) (Singleton et al., 2021). HIF-1 is a heterodimeric transcriptional factor formed of HIF-1α and a constitutively expressed HIF-1β subunit. Upon activation and nuclear translocation, HIF-1 binds to hypoxia responsive elements (HREs) in the promoter regions of target genes whose protein products mediate angiogenesis, remodeling of TME extracellular matrix, invasion, and metastasis (Singleton et al., 2021). Genome-wide association studies show that HIF-1α and its target genes are upregulated in breast cancer, and clinical data suggest that, compared to hormone responsive and HER-2 positive breast cancers, TNBCs are particularly enriched for HIF-1 and its target genes (Ma et al., 2022). Their high protein levels in primary breast cancer tumor biopsies are independently associated with poor prognostic outcomes (Ramírez-Tortosa et al., 2022; Tutzauer et al., 2022). Importantly, residual breast cancer cells surviving chemotherapy display stem-like traits (Creighton et al., 2009) and knocking down HIF-1α in these cells dampens their tumor-initiating capacity (Xiang et al., 2014). Phenotypic characteristics of CSCs are also governed by another transcriptional factor, namely, β-catenin, the activity of which is subject to upstream glycogen synthase kinase-3β (GSK-3β) modulation. Research suggests that the GSK-3β/β-catenin pathway is fundamental to the CSC program in TNBC where curbing the pathway’s activity was experimentally found to improve response to chemotherapy (Merikhian et al., 2021). Furthermore, an intriguing crosstalk between HIF-1α and β-catenin was previously described showing a synergistic interaction towards epithelial to mesenchymal transition (Zhang et al., 2013) essentially enhancing CSC behavior. These observations suggest that interference with HIF-1α/β-catenin could be a vulnerability leveraged for targeting CSCs in breast cancer. Aldehyde dehydrogenase-1 (ALDH-1), sex-determining region Y-box 2 (SOX2), and Nanog serve as paramount markers for breast CSCs, particularly within the aggressive landscape of TNBC, where they signify not only stemness but also resistance to chemotherapy. These markers are intricately linked to cellular responses under hypoxic conditions, orchestrated by HIF-1α, which further amplifies their regulatory roles in maintaining CSC characteristics (Lu et al., 2021). Furthermore, their expression is closely tied to the activation of the Wnt/β-catenin signaling pathway, highlighting a complex interplay that contributes to TNBC’s malignant phenotype and resistance to conventional therapies (Wend et al., 2010).

Equally influential to TME remodeling are the system-wide signaling cues that impinge on and modify the local signaling circuits connecting the tumor to its neighboring stromal and immune cells (Jin and Jin, 2020). One modifiable systemic factor that has garnered much attention in oncology is chronic stress that cancer patients suffer at their initial diagnosis accompanying them throughout their treatment journey and survivorship (Eckerling et al., 2021). Ample evidence indicates that stress responses, manifested by augmented activation of the sympathetic nervous system (SNS) and heightened corticosteroids release, impinge on critical tumorigenic processes as well as shape cancer response to therapy (Eckerling et al., 2021). Increased sympathetic drive induced by chronic restraint stress was found to promote angiogenesis in TNBC tumor-bearing mice (Thaker et al., 2006). Additionally, adrenergic blockade was shown to mitigate chemoresistance in multiple *in vivo* cancer models (Eng et al., 2015; Porcelli et al., 2020; Fjæstad et al., 2022). This is further substantiated by recent clinical data suggesting the use of sympatholytic drug modalities to enhance the chemotherapeutic response to anthracyclines or doxorubicin (DOX), a mainstay in TNBC regimens (Chang et al., 2023). Carvedilol (CAR), a non-selective β-blocker with additional α_1_-adrenoblocking effect, previously presented with unique anti-tumoral properties in various cancers including TNBC (Gillis et al., 2021). Additionally, CAR was shown to protect against cardiotoxicity in patients receiving trastuzumab and DOX (Nohria, 2013; Ma et al., 2019). Such observations in tandem with the paucity of evidence tying adrenaline to stress-associated alterations and its likely impact on chemoresistance in TNBC motivated the current study. We further explored whether and if the tumoricidal and CSC trait-altering CAR effects could be mediated through a modulation of the HIF-1α/β-catenin pathway in TNBC cells *in vitro* as well as in stress-promoted breast cancer model *in vivo*.

## 2 Material and methods

### 2.1 Cells and culture

The triple-negative breast cancer cells, MDA-MB-231, were cultured in complete Dulbecco’s Modified Eagle’s Medium (DMEM; Gibco, United States) supplemented with 10% fetal bovine serum (FBS; Gibco United States), and 1% penicillin/streptomycin antibiotic mixture (Gibco, United States). The cells were incubated under 37°C and 5% CO_2_ in a Heracell™ VIOS 160i Tri-Gas CO_2_ Incubator (Thermo Fisher Scientific, United States). Cells were passaged at ∼80% confluency. To investigate the effect of adrenaline (ADR) incubation on cell viability, the optical density (OD) in control untreated cells was compared to that of the ADR-incubated cells (10 µM for 6 h). For cytotoxicity assays, serial dilutions of DOX and CAR, each alone, were tested in ADR pre-incubated MDA-MB-231 cells to identify the IC50 values. This was followed by testing the serial dilutions of DOX combined with 10 and 20 µM of CAR, both pre-incubated with ADR, and comparing them to control untreated cells. As for the mammosphere assay, ADR pre-incubated cells were treated with the IC30 concentrations of DOX (0.01 µM) and CAR (3.5 µM), each alone, along with DOX IC30 combined with 10 and 20 µM of CAR, and then compared to controls with and without ADR pre-incubation. The favorable responses observed with the 20 μM concentration of CAR when combined with DOX, in both cytotoxicity and mammosphere assays, prompted further biochemical and molecular investigations. As such, protein and gene analyses were performed using IC30 of DOX and 20 µM of CAR, each alone and combined. Gene and protein assays were performed after 48 and 72 h contact with treatments, respectively.

#### 2.1.1 Cytotoxicity assay and synergy evaluation

MDA-MB-231 cells were overnight-incubated at a density of 10,000 cells/well in 96-well plates. The next day, cells were subjected to a 6-h incubation period with 10 µM of ADR. ADR pre-incubation was implemented for adrenergic stimulation in cancer cells. To determine the half-maximal inhibitory concentration (IC50), ten-fold serial dilutions (0.01–100 µM) of CAR and DOX, each alone, were then prepared and applied onto the cells. To test the impact of combining CAR to DOX, the former was added at fixed concentrations of 10 and 20 µM to the serial dilutions of the latter. The viability was assessed using 3-(4, 5-dimethylthiazol-2-yl)-2, 5-diphenyltetrazolium bromide (MTT) (SERVA, Germany) reagent, as previously described (Mosmann, 1983). Briefly, 72 h after applying treatments, MTT (5 mg/mL) was added and incubated with the cells for 2 h. DMSO was then used to solubilize the formazan crystals that developed from the reaction of MTT with viable cells. Absorbance at 570 nm was measured in control and treated cells using a microplate reader (BioTek, United States). Moreover, in order to gain insights into the effect of SNS stimulation on the viability of cancer cells, the optical densities of MDA-MB-231 cells with and without ADR pre-incubation were compared. The nature of the interaction between DOX and the two concentrations of CAR (10 and 20 µM) was explicated by calculating the combination index (C.I.), as previously described by Chou (Chou, 2010). The equation used for C.I. calculation was as follows: $C.I.=\frac{{\left(D\right)}_{1}}{{\left(Dx\right)}_{1}}+\frac{{\left(D\right)}_{2}}{{\left(Dx\right)}_{2}}$ , where (Dx)1 represents the dose of the drug D1 alone that inhibits the growth of cells by x% and (Dx)2 is the dose of the drug D2 alone that inhibits the growth of cells by x%. Herein x was chosen to be 50%. C.I.s with values < 1, = 1, and >1 indicate synergistic, additive, and antagonistic interactions, respectively.

#### 2.1.2 Mammosphere assay

MDA-MB-231 cells were seeded at a density of 3,000 cells/well in 6-well low-attachment culture plates (Greiner Bio-One International, Netherlands) with serum-free DMEM/F12 media supplemented with 1% B-27 (Gibco, United States), 10 ng/mL fibroblast growth factor (FGF; Cell Signaling, United States), and 20 ng/mL epidermal growth factor (EGF; ThermoFisher Scientific, United States). Cells were incubated with treatments for 7 days. Mammospheres were counted and pictures were captured for calculating mean diameters using ImageJ Software (NIH, United States). Additionally, the Mammosphere Formation Index (MFI) was also calculated using this formula: (number of spheres/number of seeded cells) x 100 (Palomeras et al., 2016).

#### 2.1.3 Immunocytochemistry for Nanog and ALDH-1

MDA-MB-231 cells were centrifuged using a Shandon Cytospin at speeds between 1,200 and 1,500 r/min for 15 min (Thermo Fisher Scientific, Waltham, Massachusetts), and the resulting cell pellets were spread onto glass slides. These slides were then fixed in 95% ethanol for 24 h in preparation for immunostaining. Immunostaining employed monoclonal antibodies against Nanog (Cat. no.: sc-374103, Santa Cruz Biotechnology Inc., Santa Cruz, CA, United States) and ALDH-1A1 (Cat. no.: sc-166362, Santa Cruz Biotechnology Inc., Santa Cruz, CA, United States) . After dehydration in ethanol gradients and PBS washes, slides received 200 μL of either Nanog or ALDH-1 primary antibodies (diluted 1:100) and were incubated for 4 h at 4°C in a humid chamber. Following PBS rinsing, slides were treated with a secondary biotinylated antibody for 30 min, then with an avidin-peroxidase complex for another 30 min as per the universal Detection Kit (Dako, Denmark). Development was achieved with diaminobenzidine until a brown color appeared within 5 min, followed by a distilled water wash and Mayer’s hematoxylin counterstaining for 60 sec. All steps were performed at room temperature. Negative controls in which the primary antibody was omitted and replaced by PBS were also included.

#### 2.1.4 Estimation of HIF-1α gene expression levels

HIF-1α gene expression levels were estimated using qRT-PCR in control, ADR-, ADR + DOX-, ADR + CAR-, and ADR + DOX + CAR-treated cells. RNA was isolated from MDA-MB-231 cell lysates using the Direct-zol RNA Miniprep Plus kit (Cat. no.: R2072, ZYMO RESEARCH CORP., United States). The quantity and quality of isolated RNA were evaluated using the A260/A280 absorbance ratio. Reverse transcription and PCR were performed using the SuperScript IV One-Step RT-PCR kit (Cat. no.: 12594100, Thermo Fisher Scientific, Waltham, MA, United States). Cycle threshold (Ct) values were identified for each gene. Relative expression levels were calculated using the delta-delta Ct (ΔΔCt) method, with β-actin serving as the housekeeping gene. The primer sequence of the used genes and their National Center for Biotechnology Information (NCBI) accession numbers are as follows: HIF-1α (NCBI accession no.: NM_181054.3) forward 5′- TATGAGCCAGAAGAACTTTTAGGC-3′ and reverse 5′- CACCTCTTTTGGCAAGCATCCTG-3’; β-actin (NCBI accession no.: NM_001101.5) forward 5′-CACCATTGGCAATGAGCGGTTC-3′ and reverse 5′-AGGTCTTTGCGGATGTCCACGT-3’.

#### 2.1.5 Estimation of HIF-1α and β-catenin protein levels

HIF-1α and β-catenin levels were estimated using ELISA in control, ADR-, ADR + DOX-, ADR + CAR-, and ADR + DOX + CAR-treated cell lysates following the manufacturers’ instructions of the human HIF-1α ELISA kit (Cat. no.: ab171577; Abcam, United States) and the β-catenin ELISA Kit (Cat. no.: MBS164367; MyBioSource, United States), respectively.

### 2.2 *In vivo* investigations

Female BALB/c mice, 6–7 weeks of age and weighing 20–25 g, were used for the *in vivo* experiments. Mice were kept under standard conditions of temperature, humidity, and light/dark cycles. All animal experiments were carried out in accordance with the International Guidelines for the Care and Use of Laboratory Animals and were approved by the Ethics Committee of the Faculty of Pharmacy, the British University in Egypt (EX-2210).

#### 2.2.1 Stress-promoted solid tumor animal model

**FIGURE 1:**
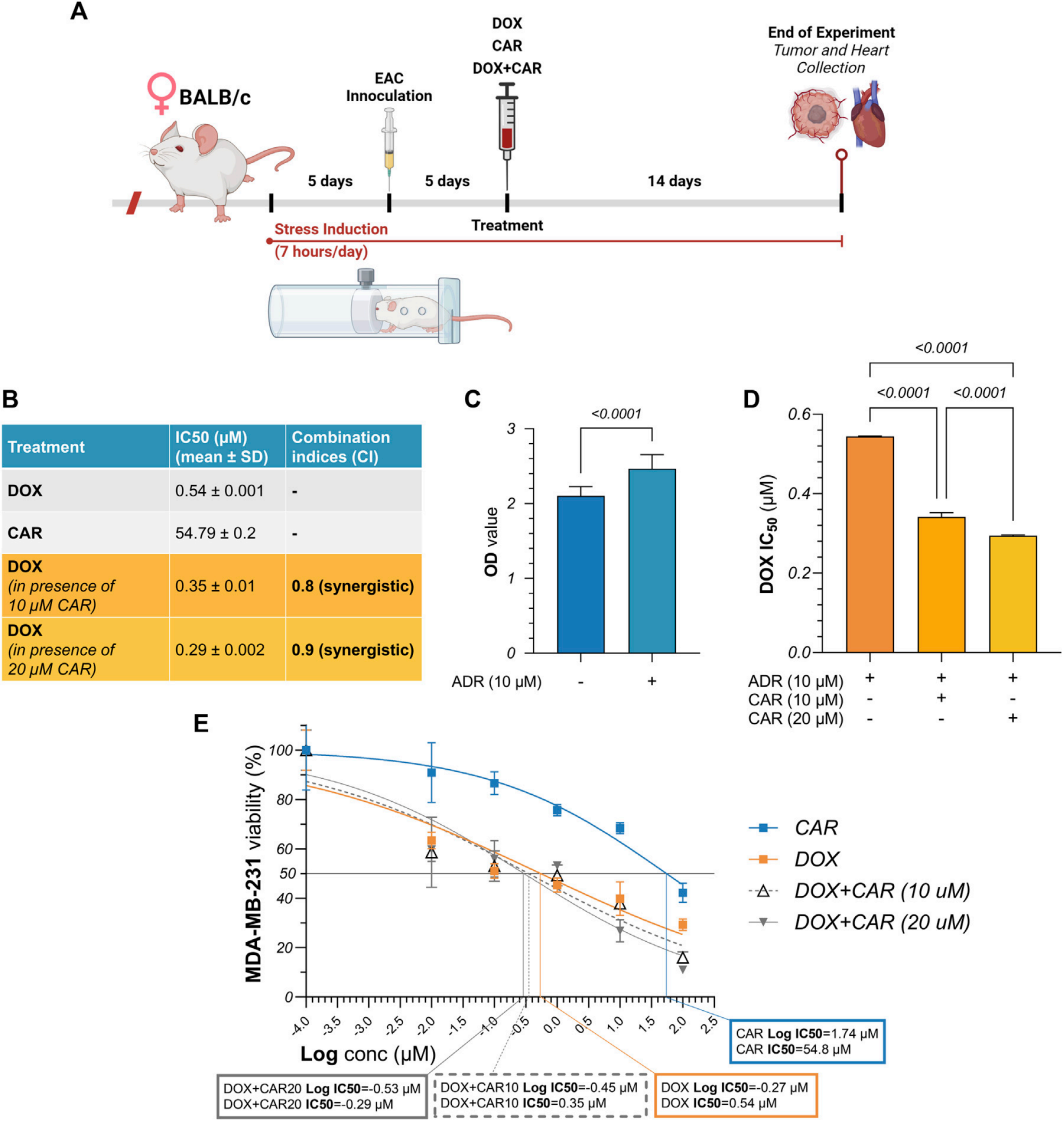
**(A)** Schematic illustration of experimental timeline for *in vivo* investigations. Female BALB/c mice were subjected to chronic restraint stress using 50 mL conical tubes, 7 h per day throughout the experimental period of 24 days. Following an initial 5-day period of stress induction, mice were inoculated with Ehrlich ascites carcinoma (EAC) cells intramuscularly into their left thighs. After another 5 days, when tumors reached palpable mass, treatment was initiated with CAR, DOX, or their combination for 14 days. Mice were sacrificed and hearts and tumors were harvested thereafter. **(B)** Summary table for the IC50 values of DOX and CAR, alone and combined, along with their combination indices (C.I.s). **(C)** Comparative analysis for the optical density measured following cell viability assay in MDA-MB-231 cells with and without ADR pre-incubation. **(D)** Comparative analysis for the IC50 values of DOX alone against its combinations with CAR in ADR pre-incubated MDA-MB-231 cells. **(E)** Dose-response curve showing the effect of CAR, DOX, and their combination on ADR pre-incubated MDA-MB-231 cell viability, as detected by MTT assay. Results are presented as means ± S. D (*n* = 3–5). Statistical difference was tested using Student’s *t*-test for unpaired data in **(C)** and one-way ANOVA followed by Tukey’s multiple comparison test in **(D)**, and significance was inferred for *p* < 0.05. IC50 values were interpolated from dose-response curves using a non-linear regression model.

A combined experimental protocol of chronic restraint stress (CS) and Ehrlich ascites carcinoma (EAC) solid tumor model, referred to herein as EAC/CS, was adopted in the current study to recapitulate stress-induced SNS activation and investigate its likely impact on tumor progression and chemoresistance (Zhang et al., 2019). All mice were subjected to restraint stress for 7 h/day, 5 days prior to EAC inoculation, and repeated daily thereafter throughout the experimental protocol. Each mouse was restrained and placed in a 50 mL conical tube with numerous holes for ventilation. Mice were then injected intramuscularly with EAC cells into their left thighs at a density of 2 × 10^6^ cells per mouse suspended in 0.2 mL saline for each. EAC tumors were then allowed to grow and reach palpable mass for 5 days before treatment initiation. Mice were randomly divided into the following groups (10 mice in each): **(I) EAC/CS**: Mice only received drug vehicles; **(II) DOX**: Mice received a single weekly dose of DOX at 4 mg/kg, administered intraperitoneally; **(III) CAR**: Mice received CAR at a dose of 10 mg/kg three times per week administered orally; **(IV) DOX + CAR**: Mice received a combination of the aforementioned treatments at their respective regimens. Another EAC-inoculated control group with no prior exposure to chronic stress (referred to as EAC) was also included for comparison with the EAC/CS group to validate the effect of chronic stress on the growth of tumors and other parameters. Additionally, to attest the cardioprotective effect of CAR, normal mice (*n* = 5) were enrolled in the experiment and received drug vehicles only to serve as a comparative group. For all groups, sacrifice was performed under anesthesia after 24 days from the start of the experiment (Figure 1A). Harvested heart and tumor tissues were either preserved in 10% formalin for histopathological evaluation or snap-frozen and stored at −80°C for subsequent biochemical analyses.

#### 2.2.2 Tumor volume, body weight, survival

Tumor dimensions were measured on days 2, 5, 8, 11, and 14 after treatment using a digital caliper. Tumor volume was then calculated using the following equation (Jaganathan et al., 2010) with the right flank size serving as an internal control for each mouse:

Tumor volume (mm^3^) = Length (mm) x Width^2^ (mm^2^) x (π/6)

Mice were weighed regularly and monitored daily to track their survival.

#### 2.2.3 Histopathological evaluation of tumors and heart tissues

Tumor and cardiac tissues were fixed in 10% formalin and subsequently processed for paraffin embedding. Thereafter, 4 μm-thick sections were sliced from the paraffin blocks using a rotary microtome (microTEC, Duisburg, Germany) and next stained with hematoxylin and eosin (H&E). Ten random fields per section per mouse were then photographed at ×40 and ×400 magnification powers using a light microscope (Zeiss, Oberkochen, Germany). Leica application computer analyzer system (Leica Microsystems, Switzerland) was used to estimate necrotic indices (NI) from the captured photomicrographs of tumor sections at x40.

#### 2.2.4 Immunohistochemical analysis for caspase-3, HIF-1α, and ALDH-1

Avidin-biotin-immunoperoxidase technique was used for immunohistochemistry (Hsu et al., 1981). Primary monoclonal antibodies with murine reactivity against caspase-3 (Cat. no.: sc-56053, Santa Cruz Biotechnology Inc., Santa Cruz, CA, United States), HIF-1α (Cat. no.: sc-13515, Santa Cruz Biotechnology Inc., Santa Cruz, CA, United States), and ALDH-1 (Cat. no.: sc-166362, Santa Cruz Biotechnology Inc., Santa Cruz, CA, United States) were used following a dilution of 1:100. For further colorimetric detection, streptavidin–biotin-peroxidase preformed complex and peroxidase-3, 3′- diaminobenzidine were used complying with the manufacturer’s instructions (Dako, Denmark). Mayer’s hematoxylin was used for counterstaining. Expression percentages were then semi-quantitatively estimated in ten random fields/section at x400 for each animal. Negative control slides, with no added antibodies, were included in each run to ensure staining specificity.

#### 2.2.5 Estimation of SOX2 gene expression in tumors

SOX2 gene expression levels were estimated using qRT-PCR in tumor tissues. RNA, reverse transcription, and PCR steps were performed as described in Section 2.1.4. With glyceraldehyde 3-phosphate dehydrogenase (GAPDH) serving as the housekeeping gene. The primer sequence of the used genes and their National Center for Biotechnology Information (NCBI) accession numbers are as follows: mouse SOX2 (NCBI accession no.: NM_011443.1) forward 5′- AAC​GGC​AGC​TAC​AGC​ATG​ATG​C-3′ and reverse 5′- CGA​GCT​GGT​CAT​GGA​GTT​GTA​C-3’; mouse GAPDH (NCBI accession no.: NM_008084.2) forward 5′- CAT​CAC​TGC​CAC​CCA​GAA​GAC​TG-3′ and reverse 5′- ATG​CCA​GTG​AGC​TTC​CCG​TTC​AG-3’.

#### 2.2.6 Estimation of caspase-3, GSK-3β, p-GSK-3β, and β-catenin protein levels in tumors

Flash-frozen tumor tissues were homogenized in ice-cold phosphate buffered saline (PBS; Lonza, Switzerland) as 10% weight/volume. Total protein content was estimated using Pierce™ BCA Protein Assay Kit (Cat. no.: 23225; ThermoFisher Scientific, United States). Caspase-3 (MyBioSource, United States), phosphorylated (serine-9) GSK-3β (R&D Systems, United States), and total GSK-3β (BioVision, United States) as well as β-catenin (MyBioSource, United States) were then estimated in tumor homogenates using ELISA kits following the respective manufacturers’ instructions.

### 2.3 Statistical analysis

Data were compiled, visualized, and tested for statistical difference using GraphPad Prism software V9.0 (GraphPad, Inc., United States). Results were expressed as mean ± standard deviation. Survival analysis was tested using Kaplan-Meier test, while other parameters were tested using Student’s *t*-test or one-way ANOVA followed by Tukey post-hoc test for multiple comparisons. IC50 values were interpolated from dose-response curves using a non-linear regression model. Tumor volume data were log-transformed following Brown–Forsythe test. Significant difference was inferred for *p* values below a threshold value of 0.05.

## 3 Results

### 3.1 CAR synergizes with DOX in promoting cytotoxicity of ADR pre-incubated MDA-MB-231 cells

ADR is one of the most extensively studied neurotransmitters within the setting of stress-promoted cancer. To recapitulate its impact on TNBC proliferation *in vitro* (Figures 1A-E), MDA-MB-231 cells were incubated with ADR for 6 h and viability was compared to that of their untreated counterparts. We found that ADR caused a 1.2-fold surge in cell viability compared to untreated cells suggesting a proliferative role for adrenergic signaling in TNBC (Figure 1C). We next exposed ADR pre-incubated cells to increasing concentrations of DOX and CAR, each alone, to estimate the IC50 of each treatment in the presence of ADR. As shown in the summary table in Figure 1, DOX and CAR had a mean IC50 of 0.54 and 54.79 µM, respectively. Combining CAR at a concentration of 10 and 20 µM to DOX elicited reductions in cell viability reaching 35% (IC50 = 0.35 µM) and 46% (IC50 = 0.29 µM) with CIs of 0.8 and 0.9, respectively (Figures 1D,E). These results, therefore, suggest a synergistic mode of interaction between CAR and DOX in MDA-MB-231 TNBC cells.

### 3.2 CAR and DOX combination impacts mammosphere population in ADR pre-incubated MDA-MB-231 cells

**FIGURE 2:**
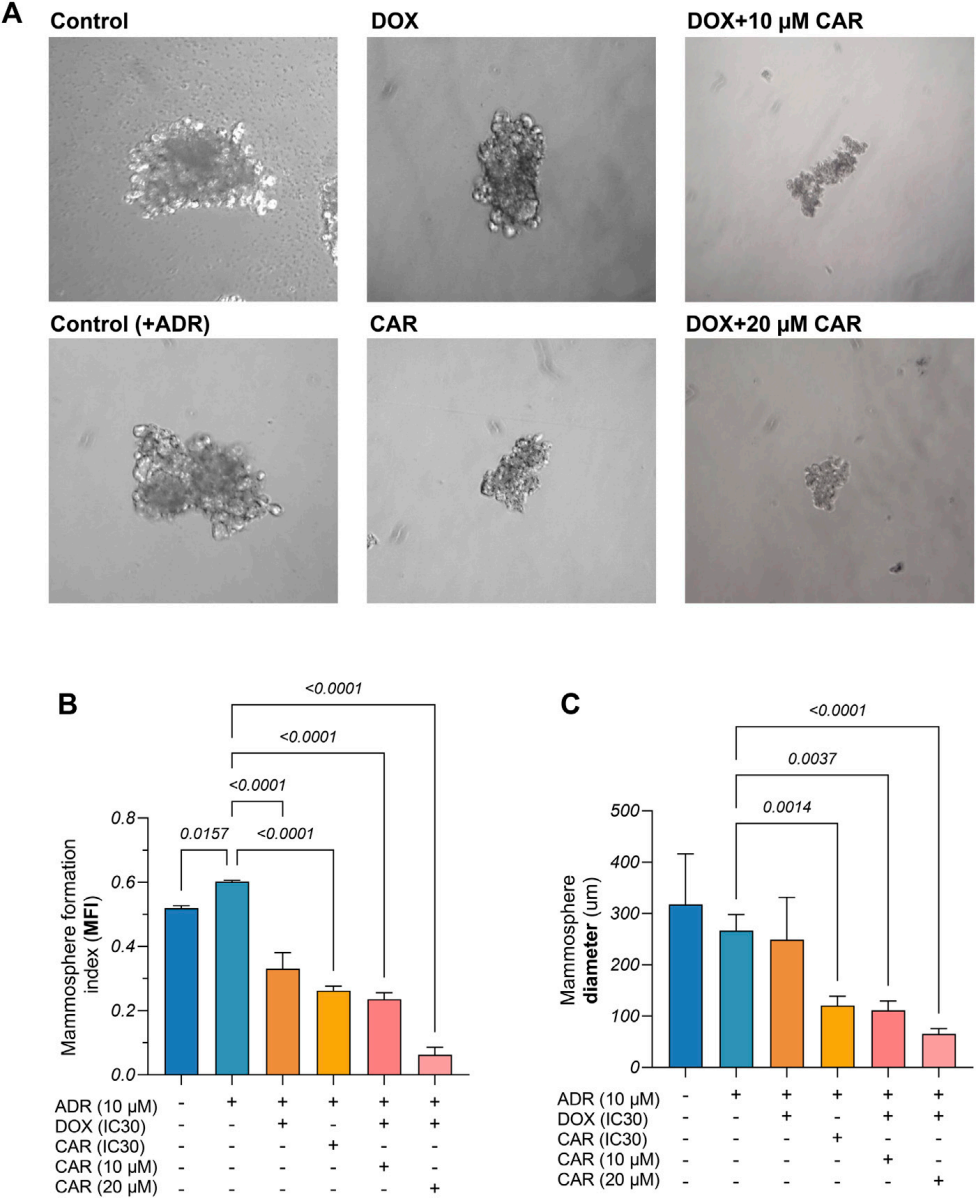
**(A)** Representative images of mammospheres derived from control and adrenaline (ADR) pre-incubated MDA-MB-231 cells and those exposed to CAR, DOX, or their combination. **(B)** Mammosphere formation index (MFI%) as well as **(C)** Mammosphere diameters from different control and treated MDA-MB-231 cells. Results are presented as means ± S. D (*n* = 3). Statistical difference was tested using one-way ANOVA, followed by Tukey’s multiple comparison test, and significance was inferred for *p* < 0.05.

In order to investigate the likely impact of ADR in promoting stemness traits in TNBC and whether adrenergic blockade by CAR can mitigate this effect, mammosphere assay was conducted and two indicators were used, namely, MFI (%) and mammosphere diameter (Figure 2A). For the MFI, ADR-incubated MDA-MB-231 cells showed a 1.2-fold higher MFI% than ADR-untreated cells. Moreover, DOX and CAR demonstrated 55% and 88% reductions in MFI compared to ADR-incubated control cells, respectively. Combining DOX to 10 and 20 µM of CAR showed 29% and 81% reductions in MFI compared to DOX-treated cells, respectively. It is noteworthy that the latter combination exhibited the lowest MFI% among other treatments (Figure 2B). Regarding the mammosphere diameters, the ADR-incubated cells showed no difference compared to their untreated counterparts. Also, DOX did not cause a change in the mean diameter compared to ADR-treated control cells. Treatment with CAR, however, exhibited a 55% reduction in mammosphere diameter compared to ADR-incubated control. Moreover, combining CAR to DOX at 10 and 20 µM concentrations demonstrated 55% and 74% reductions, respectively, as compared to DOX monotherapy (Figure 2C).

### 3.3 CAR and DOX combination countervailed the CSC markers, ALDH-1 and Nanog, in ADR pre-incubated MDA-MB-231 cells

**FIGURE 3:**
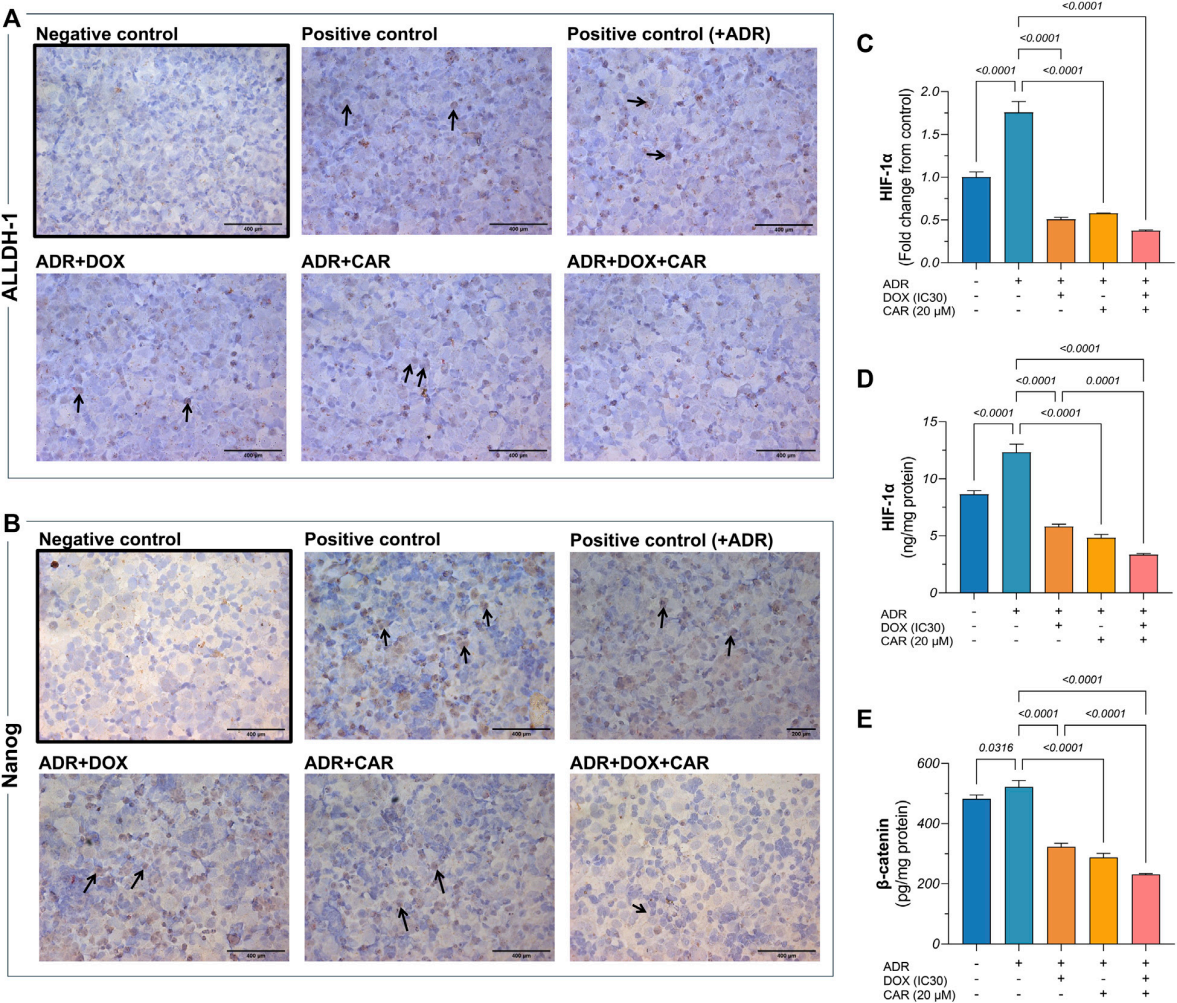
Representative photomicrographs (DAB, x400) for immunocytochemical analysis against **(A)** ALDH-1 and **(B)** Nanog in MDA-MB-231 cells including those pre-incubated with ADR and subjected to different treatments, as well as ADR untreated cells. Gene expression of **(C)** HIF-1α as well as protein levels of **(D)** HIF-1α and **(E)** β-catenin, as estimated by qRT-PCR and ELISA, respectively. Immunocytochemical positive expression appears as brown cytoplasmic and nuclear discoloration for ALDH-1 and Nanong, respectively, as indicated by the black arrows. Results are presented as means ± S. D (*n* = 5). Statistical difference was tested using one-way ANOVA, followed by Tukey’s multiple comparison test, and significance was inferred for *p* < 0.05.

ALDH-1 and Nanog play pivotal roles in maintaining the stemness of TNBC cells by promoting self-renewal, suppressing differentiation, and enhancing tumorigenic potential, to foster its aggressive nature. This insight prompted the investigation of the effect of ADR on ALDH-1 and Nanog expression through immunocytochemical analysis. Our observations revealed marked ALDH-1 and Nanog expression in ADR-treated cells in contrast to moderate expression in untreated controls (Figures 3A,B). Additionally, DOX treatment resulted in moderate expression levels of ALDH-1 and Nanog, whereas CAR exhibited mild expression for both markers. Notably, the DOX+CAR combination yielded scant positive cells, nearing negative expression in certain instances for both markers. These findings suggest that DOX and CAR combination might interfere with CSC maintenance and their self-renewal capabilities.

### 3.4 CAR and DOX combination curbed HIF-1α on both gene and protein levels and decreased β-catenin protein levels in ADR pre-incubated MDA-MB-231 cells

HIF-1α is pivotal in modulating CSC dynamics within TNBC, steering hypoxic adaptations that enhance stemness and therapeutic resistance, thus significantly contributing to TNBC’s aggressive behavior. This regulatory mechanism seamlessly integrates with the Wnt/β-catenin pathway, further amplifying CSC proliferation and differentiation blockade, thereby underpinning a key axis in TNBC resistance (Sulaiman et al., 2020). Accordingly, exploring the changes in the levels of these two markers was sought to reflect on how the combination of DOX and CAR, under stress-simulated conditions by ADR pre-incubation, can impact this signaling network. As shown in Figures 3C,D, ADR caused a 1.8- and 1.4-fold increase in HIF-1α gene expression and protein levels, respectively, compared to control untreated cells. Moreover, DOX, CAR, and their combination elicited 71, 67, and 79% reductions in HIF-1α gene expression levels in ADR pre-incubated cells, respectively, as compared to their untreated counterparts. Likewise, treatment with DOX, CAR, and their combination caused reductions in HIF-1α protein levels in ADR pre-incubated cells reaching 53, 61, and 73%, respectively, when compared to ADR untreated control cells.

In a similar vein, ADR demonstrated an increase in β-catenin levels compared to control cells confirming the role of SNS activation in upregulating the Wnt/β-catenin pathway in TNBC (Figure 3E). Additionally, DOX, CAR, and their combination demonstrated 38, 44, and 56% reduction in β-catenin levels in ADR pre-incubated MDA-MB-231 cells compared to their untreated controls. It is noteworthy that the combination caused an almost 30% reduction in β-catenin levels, as compared to DOX alone.

### 3.5 Effect of CAR and DOX combination on tumor volumes, survival, and body weight

**FIGURE 4:**
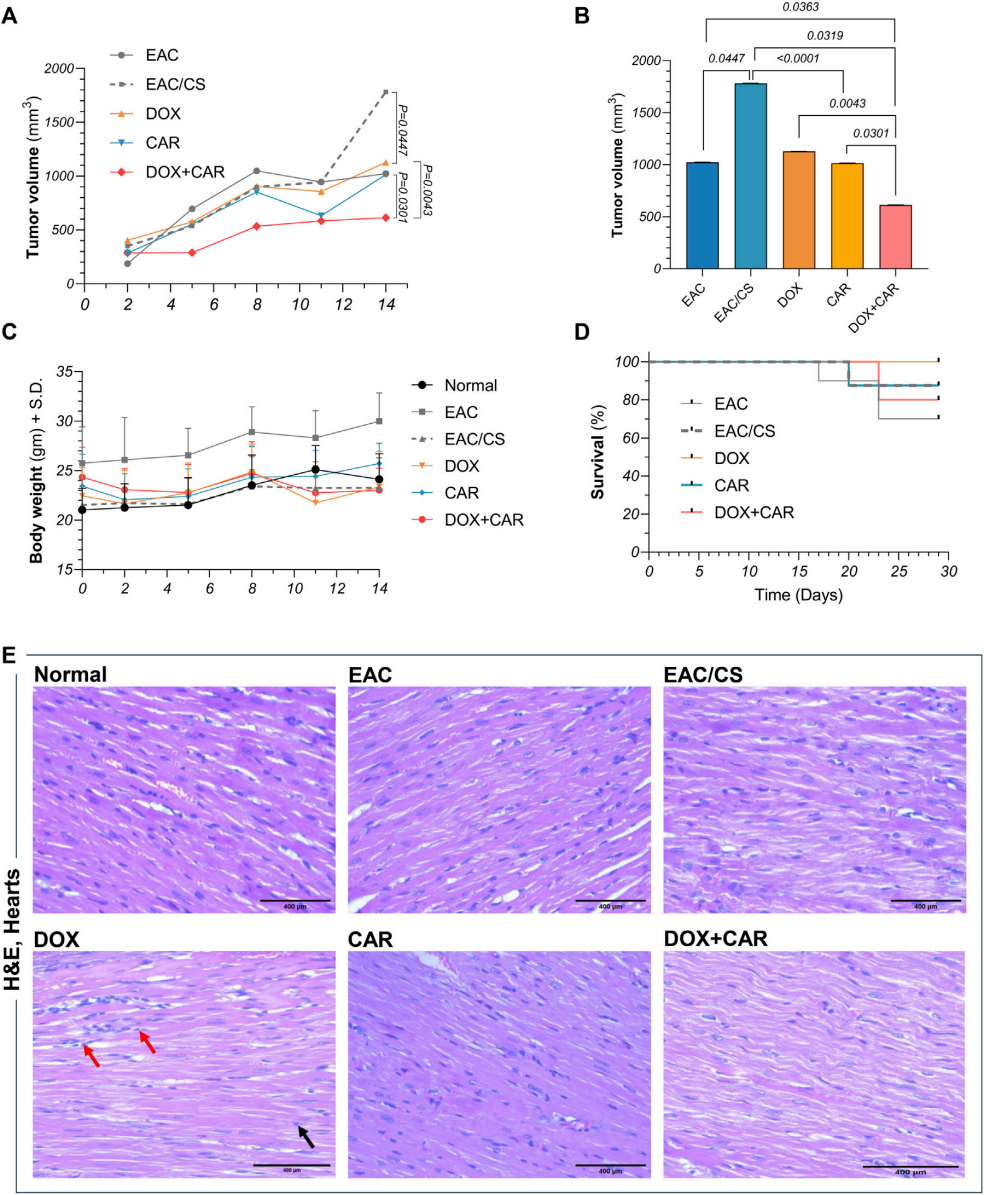
**(A)**Tumor volume (mm^3^) in EAC-bearing mice at different time points across different experimental groups. **(B)** Comparative analysis of the mean tumor volumes at day 14 post-treatment, as well as **(C)** body weights and **(D)** survival curves for control and treated groups at different time points.**(E)** Representative photomicrographs showing the histopathological changes in H&E-stained heart sections (x400) from control and treated groups. DOX-treated group showing focal areas of widely separated cardiomyocytes with intercellular inflammatory cell infiltrates (red arrow). Many cardiac cells demonstrated fragmentation of myofibrils with or without pyknotic nuclei (black arrow). Values are presented as means ± S. D (*n* = 6–8). Tumor volume data underwent log transformation and tested for statistical significance using one-way ANOVA followed by Tukey’s multiple comparison test. Survival data was analyzed using Kaplan Meier test. Significance was inferred for *p* values below 0.05 critical threshold level.

To explore the impact of CAR on DOX's therapeutic response in stress-promoted cancer, tumor volumes were estimated at different time points during the experiment and just before sacrifice. As shown in Figures 4A,B, combining CAR to DOX resulted in almost 45% reduction in tumor size compared to DOX alone at day 14 post-treatment. It is noteworthy that the EAC/CS group elicited a 1.8-fold increase in tumor volume compared to the EAC group. Such a finding suggests that the milieu of chronic stress may exacerbate tumor progression. Consequently, this observation prompted the use of EAC/CS as a pivotal reference point for evaluating the efficacy of various treatments in subsequent investigations.

In order to assess the overall safety of the treatment regimen, mice were weighed regularly throughout the experiment and monitored daily for changes in vitality and excitability. Mortality was regularly recorded as well. As shown in Figure 4C, there were no significant alterations observed in body weights. On the other hand, despite the mortality encountered in some of the experimental groups, no significant change in survival was observed (Figure 4D), suggesting the safety profiles of these therapeutic modalities when used in combination at the tested dose levels.

### 3.6 CAR protects against DOX-induced cardiotoxicity

The effect of DOX, CAR, and their combination on the hearts of mice was examined by H&E staining and shown in Figure 4E. H&E-stained cardiac sections of DOX-treated group depicted focal areas of necrotic cardiomyocyte dissociation with interstitial inflammatory infiltrates. Moreover, many cardiomyocytes showed myofibrillar disarray with or without pyknosis. CAR-treated group, however, showed apparently normal cardiomyocytes without signs of inflammation. On the other hand, DOX + CAR group displayed more preserved cardiac muscle tissue architecture as compared to DOX, as evidenced by cardiomyocytes interspersed with fewer numbers of degenerated cells. Milder inflammatory cells infiltration was also observed suggesting a protective role for CAR against DOX-induced cardiotoxicity. The protective effect exhibited by the combination in cardiac tissue sections paralleled that observed in sections from healthy hearts of normal mice. Moreover, it is worth mentioning that H&E-stained sections from EAC and EAC/CS groups depicted no insults to cardiac tissues.

### 3.7 CAR and DOX combination improves tumor histopathology and promotes necrosis in solid tumors

**FIGURE 5:**
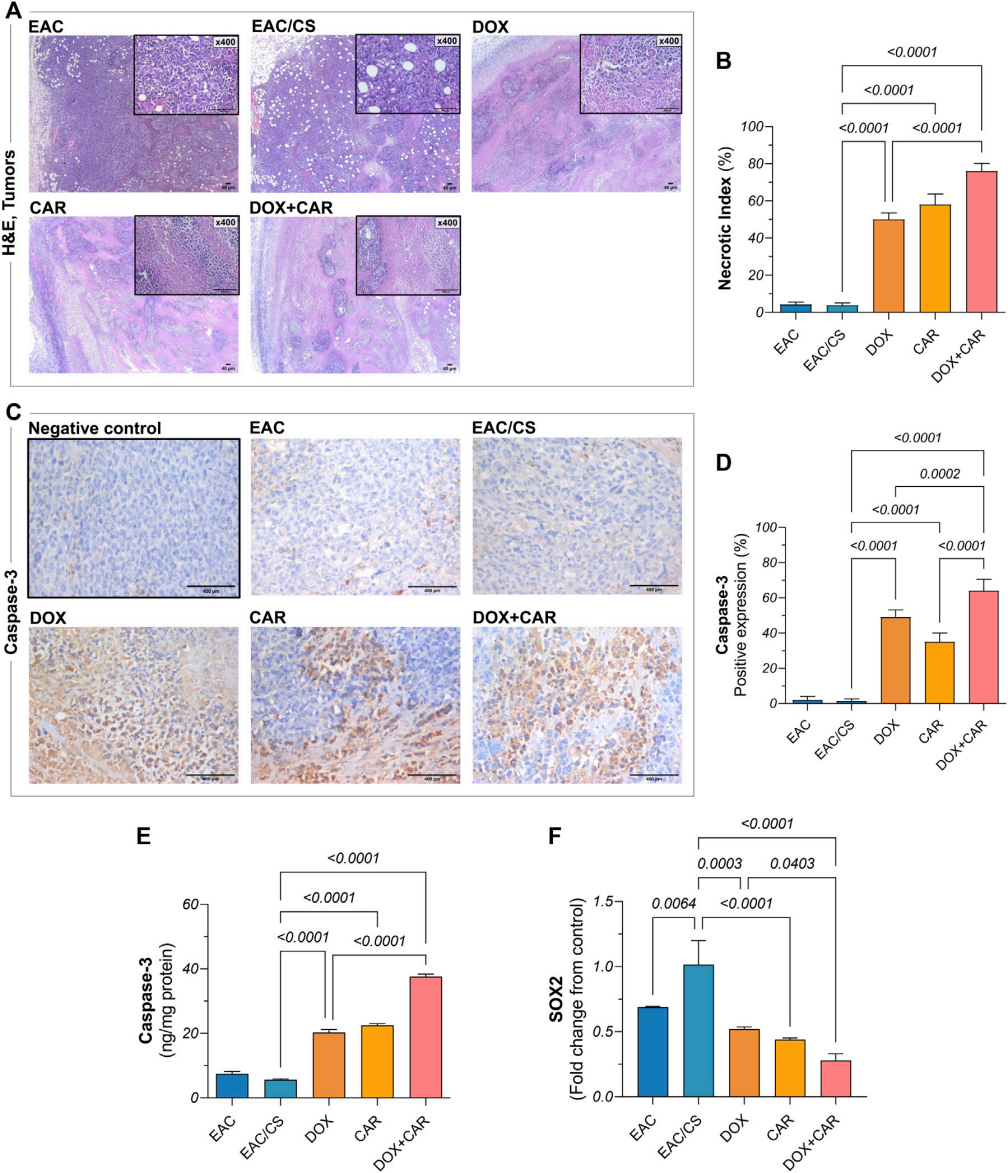
**(A)** Representative photomicrographs showing histopathological alterations in H&E-stained tumor sections (x40, x400) from EAC-bearing mice in control and treated groups and corresponding bar chart for **(B)** necrotic indices. **(C)** Representative photomicrographs of anti-caspase-3 immunohistochemical staining (DAB, x400) of tumor tissue sections of EAC-bearing mice belonging to control and treated groups and their **(D)** percentage of positive expression estimated semi-quantitatively. **(E)** Caspase-3 protein levels and **(F)** SOX2 gene expression, as estimated in tumor tissues using ELISA and qRT-PCR, respectively. Results are presented as means ± S. D (*n* = 5). Statistical difference was tested using one-way ANOVA, followed by Tukey’s multiple comparison test, and significance was inferred for *p* < 0.05.

Histopathological evaluation was performed on H&E-stained tumor sections in order to investigate the malignant histological changes and to provide insights into the capacity of treatments on modulating these alterations under stress-promoted conditions. As shown in Figure 5A, both untreated controls, EAC and EAC/CS, depicted large, irregular, and densely packed tumor cells with abundant cytoplasm and prominent nuclei, however, the EAC/CS group showed more heightened malignant features compared to the EAC group. In contrast, DOX-treated sections showed reduced tumor cellularity, with immune infiltration and large areas of necrosis, the latter was reflected in a 13-fold increase in necrotic index, as compared to untreated control (Figure 5B). The CAR group also displayed smaller and less dense tumors, with less nuclear atypia and a comparable yet significant 15-fold surge in necrotic index. The combination group had the most pronounced anti-tumor effect, showing fewer malignant cells and extensive necrosis with a 1.5-fold further increase in necrotic index, as compared to the DOX-treated group.

To explore the mechanism underlying tumor cell death, immunohistochemical analysis was carried out for caspase-3, the expression of which mediates the execution phase of apoptosis. As shown in Figure 5C, the untreated group demonstrated mild caspase-3 expression, indicating tamed apoptotic activity. The DOX- and CAR-treated groups, on the other hand, enhanced caspase-3 immunohistochemical expression in tumors reaching 35% and 25%, respectively, compared to EAC/CS, reflecting their respective capacities for apoptosis induction (Figure 5D). Notably, the combination group had the highest caspase-3 expression levels reaching up to 45-fold and 1.3-fold compared to control and DOX-treated groups, respectively. To confirm the present findings, caspase-3 tumor levels were further estimated using ELISA where a nearly similar pattern was observed. Both DOX and CAR single treatments caused almost a 4-fold increase in caspase-3 levels compared to the EAC/CS control group. Meanwhile, DOX + CAR caused a 7- and 2-fold increase in caspase-3 levels compared to the EAC/CS and DOX-treated groups, respectively (Figure 5E). These findings suggest that the combined use of DOX and CAR may substantially enhance apoptosis in tumor cells.

It is worth noting that there were no significant differences observed in the necrotic indices or caspase-3 levels between the EAC and EAC/CS groups. This lack of variation can be attributed to the context-specific nature of necrosis and apoptosis in these experimental conditions, where significant alterations in these parameters are not anticipated in absence of treatment.

### 3.8 CAR and DOX combination curbs ALDH-1 and SOX2 levels as well as HIF-1α tumor expression

**FIGURE 6:**
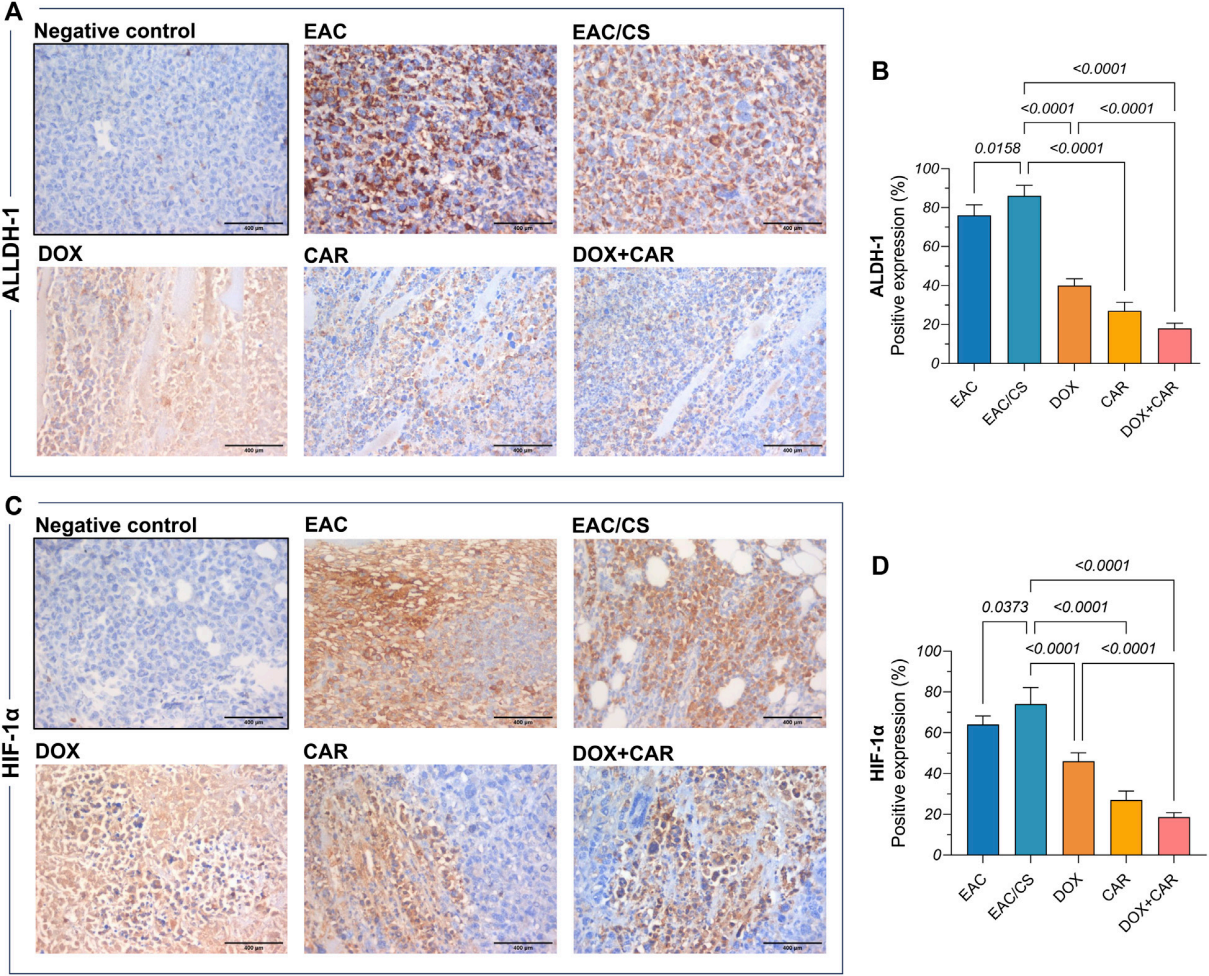
Representative photomicrographs (DAB, x400) of immunohistochemical expression against **(A)** ALDH-1 and its corresponding **(B)** percentage of positive expression along with **(C)** HIF-1α and its corresponding **(D)** percentage of positive expression. Positive expression was estimated semi-quantitatively. Results are presented as means ± S. D (*n* = 5). Statistical difference was tested using one-way ANOVA, followed by Tukey’s multiple comparison test, and significance was inferred for *p* < 0.05.

Phenotypic and functional CSC traits are regulated by the transcriptional factor HIF-1α and manifested, in part, by ALDH-1 expression, the latter serving as a surrogate indicator of stemness. Additionally, a partnership exists between SOX2, another key CSC marker, and HIF-1α to strengthen the CSC inherent stemness features. Accordingly, this has spurred the exploration of changes in ALDH-1, SOX2, and HIF-1α in tumor tissues. As shown in Figure 5F, DOX, CAR, and their combination downregulated SOX2 gene expression levels by 49, 57, and 73%, as compared to EAC/CS group. ALDH-1 immunoreactivity, on the other hand, was 54, 69, and 79% lower in the DOX-, CAR-, and DOX + CAR-treated groups than that observed in EAC/CS control group. Additionally, DOX and CAR combination was superior to DOX monotherapy in decreasing ALDH-1 expression levels by 55% (Figures 6A,B). Similarly, Figures 6C,D demonstrate that DOX, CAR, and their combination caused 38, 64, and 75% reductions in HIF-1α expression compared to EAC/CS untreated group. Moreover, the group that received DOX and CAR combination depicted a further 60% reduction compared to DOX monotherapy.

Remarkably, the EAC/CS group exhibited a 1.5-fold increase in SOX2 levels, along with a 1.13-fold increase in ALDH-1, and a 1.2-fold increase in HIF-1α levels, when compared to the EAC group. These results might imply a heightened enrichment in the CSC population within a potentially hypoxic niche in the group exposed to chronic stress.

### 3.9 CAR and DOX combination modulates GSK-3β/β-catenin signaling *in vivo*

**FIGURE 7:**
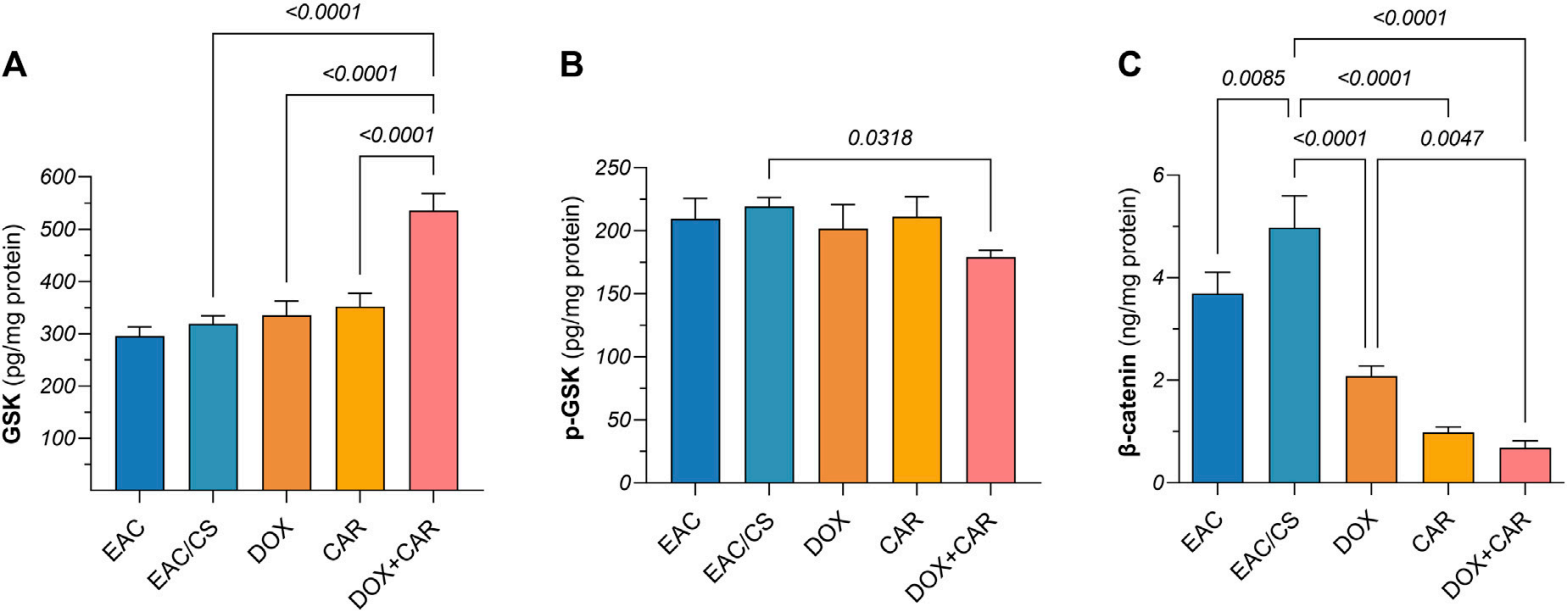
Protein levels of **(A)** GSK-3β, **(B)** p-GSK-3β, and **(C)** β-catenin in tumor tissue homogenates as estimated by ELISA after normalization to protein content. Results are presented as means ± S. D (*n* = 5). Statistical difference was tested using one-way ANOVA, followed by Tukey’s multiple comparison test, and significance was inferred for *p* < 0.05.

To gain further mechanistic insights into the molecular underpinnings mediating the effect of CAR on CSC-related traits and how adrenergic blockade modulates the tumoral response towards DOX chemotherapy in chronic stress settings, protein levels of β-catenin and its upstream modulator, GSK-3β, were estimated in tumor tissues. As shown in Figures 7A,B, neither DOX nor CAR single treatments resulted in significant changes in GSK-3β or p (S9)- GSK-3β protein levels. On the other hand, combining CAR to DOX resulted in a significant 1.6-fold upsurge in GSK-3β and an 11% reduction in p-GSK-3β compared to DOX-treated mice. Moreover, combining CAR to DOX resulted in the highest suppression reaching 86% and 67% in β-catenin levels compared to EAC/CS control and DOX groups, respectively (Figure 7C). It is also worth mentioning that β-catenin levels in the EAC/CS group was 1.4-fold higher than that observed with EAC. These results indicate that adjuvant use of CAR with DOX might modulate the key stem cell GSK-3β/β-catenin pathway in chronic stress promoted cancer.

## 4 Discussion

Chemoresistance remains a major hurdle towards successful eradication of TNBC with accumulating evidence depicting an essential role for CSCs in therapeutic failure and tumor recurrence (He et al., 2021). Cancer patients are afflicted with enormous stress that is entangled to their cancer diagnosis and their exposure to cycles of systemic chemotherapies. Experimental evidence supports the notion that stress-induced sympathetic activation not only virtually fuels almost all the recognized hallmarks of cancer but also lays the foundation for fostering resistance to therapy (Eckerling et al., 2021). Research endeavors have particularly placed emphasis on the immunomodulatory effects of adrenergic signaling in the TME (Liu et al., 2022). In the current study, we therefore sought to untangle the mechanistic links tying adrenaline signaling to TME elements that shape CSCs behavior in their hypoxic niche, integrating stress-associated changes that modify their enrichment and by extension, whether and how adrenergic blockade using CAR could be leveraged to sensitize TNBC cells towards DOX treatment. This hypothesis was further explored in an *in vivo* EAC solid tumor animal model in which mice were pre-exposed to chronic stress before and after inoculation. Here, we aimed to ascertain the influence of the hypoxic niche within tumors on the behavior of CSCs and to explore the potential implication of the GSK-3β/β-catenin signaling pathway in mediating these effects within the stressed TME.

To this end, we first tested our hypothesis *in vitro* using the TNBC cell line, MDA-MB-231, in the presence of ADR, thus mimicking stress-induced SNS activation. Cytotoxicity studies showed that the viability of TNBC cells was significantly higher when pre-incubated with ADR suggesting a proliferative role for adrenergic signaling. Combining CAR to DOX was also found to enhance the latter’s cytotoxicity in ADR-preincubated MDA-MB-231 cells. These effects could mostly be attributed to the β-adrenergic blockade of CAR. Compared to other breast cancer cell lines, MDA-MB-231 exhibit the highest expression of β-adrenoreceptors (Jeong et al., 2022) and treating these cells with ADR confers resistance to apoptosis through BAD protein inhibition (Sastry et al., 2007). Moreover, β-adrenergic blockade using propranolol curbed the levels of proliferative cyclin proteins in part by inducing the stabilization and subsequent activation of the tumor-suppressor p53 (Montoya et al., 2019). Our results are also consistent with previous studies leveraging sympatholytic drugs to enhance the sensitivity of breast cancer cells when used either alone or in combination with chemotherapeutic agents like trastuzumab (Rico et al., 2016; Liu et al., 2016).

The capacity of tumor-derived cancer cells to form spheroids is a conventional screening tool to test for functional traits reminiscent of CSCs (Nath and Devi, 2016). Previous evidence indicated that ADR supplementation to the culture media enhanced spheroid formation in multiple cell lines. For instance, ADR-treated colorectal cancer cells form larger spheroids than their control counterparts (Zhou et al., 2022). Similarly, tumor cells from stressed nude-mice xenografted with MDA-MB-231 cells were found to form mammospheres more efficiently than their control stress-free littermates (Cui et al., 2019). Intriguingly, this study showed that exogenously administered ADR could fully recapitulate stress-induced enhancement in CSC traits. Mechanistically, the authors revealed that the ADR-activated β_2_-adrenoreceptors, expressed by TNBC cells, metabolically rewired tumoral cells towards boosted production of lactate which, in turn, led to activation of SLUG and promoted β-catenin stabilization thus enriching stem-like behavior (Cui et al., 2019). On the other hand, hormone sensitive MCF-7 and TNBC MDA-MB-231 cells exposed to DOX were found to be enriched for CSCs showing higher expression of a variety of CSC markers suggesting a putative role for this distinct subpopulation of cells in DOX chemoresistance (Doublier et al., 2012; Ozcelikkale et al., 2017; Liu et al., 2019; Paramanantham et al., 2021), a finding that was further substantiated clinically (Li et al., 2020). In concert with these observations, we found that DOX treatment resulted in a similar induction of CSC behavior when these cells were incubated with ADR which was mitigated upon CAR treatment particularly evident by the curtailment observed in MFI and mammosphere diameters along with the expression of the CSC markers, ALDH-1 and Nanog. Furthermore, adrenergic blockade alone significantly countervailed mammosphere formation independent of DOX treatment. This finding was given credence by two recent studies in hormone responsive MCF-7 breast cancer cells (Bonuccelli et al., 2022) and uveal melanoma cells where a similar concentration of CAR was shown to curb spheroid formation (Farhoumand et al., 2022). Intriguingly, these effects appear to be peculiar to CAR as other non-specific adrenergic blockers did not affect spheroid formation or tangibly did at much higher concentrations (Farhoumand et al., 2022). Such observations might allude to a molecule-specific property of CAR which still warrants further investigation.

Coordinate expression of HIF-1α and β-catenin has been shown to play a profound role in TNBC tumorigenesis and their transcriptional activity is associated with cancer relapse and poor survival (Merikhian et al., 2021). These transcriptional factors also govern the expression of multiple genes that enrich tumoral stemness (Xiang et al., 2014; Merikhian et al., 2021). Moreover, previous evidence suggests an interdependence between these transcriptional factors towards driving tumorigenesis and curbing response to therapy (Merikhian et al., 2021). Meanwhile, a regulatory crosstalk between adrenergic signaling and both HIF-1α (Hu et al., 2010; Shan et al., 2013; Wu et al., 2016; Huang et al., 2019) as well as the Wnt/β-catenin pathway (Lin et al., 2020; Liu et al., 2020) had been previously described whereby the former was shown to regulate both the stability as well as the transcriptional activity of the latter proteins. This has prompted us to hypothesize that stress-induced adrenergic activation could augment phenotypic tumoral alterations manifesting CSC traits mediated by a modulation of the crosstalk between HIF-1α and the β-catenin pathway. Our findings enrich the dialogue established by prior research as illustrated in the notable reduction of HIF-1α and β-catenin levels through adrenergic blockade by CAR in ADR pre-incubated MDA-MB-231 cells. This evidence strengthens the proposition that stress-induced adrenergic activation modulates tumor phenotype, particularly through HIF-1α and the Wnt/β-catenin pathway, thereby potentiating CSC-driven resistance to DOX. Simultaneously, this favorable impact was also evidenced by a marked decrease in ALDH-1 and SOX2 expression in tumors from mice receiving the combination therapy, affirming the interconnected roles of adrenergic blockade, CSC modulation, and chemotherapeutic resistance.

To ascertain the influence of chronic stress on tumor progression *in vivo*, we conducted a comprehensive comparison of various parameters related to tumor progression and chemoresistance, including tumor volume, histopathology, and markers indicative of CSC enrichment such as SOX2 and ALDH-1, as well as those associated with the hypoxic microenvironment such as HIF-1α, and the underlying β-catenin levels between EAC and EAC/CS groups. Across all these dimensions, our findings consistently revealed heightened tumor progression and augmented expression of CSC markers, accompanied by elevated β-catenin levels, in the group subjected to chronic stress (EAC/CS) compared to their non-stressed counterparts.

Additionally, in the present study, we found that adrenergic blockade using CAR not only ground tumor growth to a halt, as evidenced by significantly lower tumor volumes compared to control littermates before sacrifice but also resulted in tumor cell death with significantly enhanced necrosis and apoptosis, as evidenced by heightened necrotic index observed in H&E-stained tumor sections paralleling an upsurge in the pro-apoptotic caspase-3 protein levels, respectively. Our findings are in line with recent investigations whereby adrenergic blockade was associated with decreased tumor burden and increased caspase-3 levels in TNBC xenografted stressed mice (Cui et al., 2019) as well as EAC-tumor bearing mice (Zidan et al., 2023). This increase in caspase-3 levels could be explained, at least in part, by the suppressive effect CAR had on tumoral HIF-1α levels whose gene silencing results in an increase in caspase-3 levels (Cheng et al., 2016). Furthermore, combining CAR to DOX resulted in the lowest reduction in tumor volume and the highest significant increase in both necrotic indices observed in tumor tissue sections and caspase-3 levels in tumor tissue homogenates. A bi-directional relationship between chemotherapy and adrenergic signaling was described by Chang et al. who reported upregulation of β-adrenoreceptors in breast cancer cells exposed to DOX and in resected tumors of breast cancer patients receiving neoadjuvant chemotherapy suggesting that DOX could reciprocally sensitize the tumor towards ADR signaling (Chang et al., 2021). This might therefore explain CAR's enhancement of DOX cytotoxicity, in the current study, upending its resistance in stress-promoted breast cancer.

The stability of β-catenin is under the tight control of a multimeric destruction complex that includes GSK-3β which acts as a negative regulator. In contrast, phosphorylation of GSK-3β at S9 curbs its activity subsequently stabilizing β-catenin and increasing its cytoplasmic levels (McCubrey et al., 2014). In the current study, we found that β-catenin level significantly decreased in DOX mice co-treated with CAR relative to DOX alone while no significant changes were detected in p-GSK-3β levels. One plausible explanation is that β-catenin expression is not solely dictated by GSK suggesting that other modulators might be at play. In the setting of stress-promoted carcinogenesis, the Y box binding protein-1 (YB-1) was previously reported to link adrenergic signaling to β-catenin mediated chemoresistance in hepatocellular carcinoma (Liu et al., 2020) and pancreatic cancer (Shan et al., 2013). YB-1 is a DNA/RNA binding protein that has emerged as a critical regulatory node supporting tumor survival and inducing CSC enrichment (Yang et al., 2019). Silencing YB-1 on the other hand was shown to inhibit TNBC proliferation in a HIF-1α dependent manner (Lefort et al., 2022). However, whether the suppression observed in tumoral β-catenin levels in stressed mice subjected to CAR is a direct consequence of HIF-1α downregulation or mediated through YB-1 in TNBC remains an open question delineating from the current investigation. To answer this, one could consider the use of pharmacological modulators of HIF/genetic manipulation *in vitro* and subsequently estimate β-catenin activity, the contribution of which in stress-promoted anthracycline chemoresistance might plausibly be associated with CSC phenotypic changes.

In this study, the adrenergic blocker, CAR, was deliberately chosen for evaluation in conjunction with DOX, and not other chemotherapeutic agents used for the management of TNBC such as paclitaxel. This strategic selection was anchored in a constellation of critical factors. CAR’s distinctive antioxidant and anti-inflammatory attributes not only propose a comprehensive strategy to mitigate the cardiotoxic effects frequently associated with DOX (Dandona et al., 2007) but also potentiate its antineoplastic activity. Notably, the cardioprotective synergy between CAR and DOX has been substantiated in clinical realms (Tashakori Beheshti et al., 2016) and corroborated by our data, piquing our interest in elucidating the molecular mechanisms through which CAR may counteract DOX resistance and amplify its efficacy and safety profiles towards achieving better therapeutic outcomes in clinical settings.

The selection of a model that more accurately reflects the disease in question is not merely a technical detail, however, it is a critical step towards achieving a deeper understanding of a particular disease setting and, ultimately, the development of effective treatments. The EAC solid tumor model provides a platform for studying interactions between tumor cells and the tumor microenvironment, including angiogenesis, hypoxia, and immune cell infiltration. This aspect is particularly relevant to our research focus on elucidating the role of HIF-1α in tumor progression and response to therapy, as the tumor microenvironment significantly influences HIF-1α activity and downstream signaling pathways. While the 4T1 mammary intraductal model (MIND) stands out for its ability to closely recapitulate the pathological features of human TNBC (Ghosh et al., 2018), its specificity to mammary carcinoma limits its broader applicability across different cancer types. In contrast, the EAC solid tumor represents a non-specific model, and hence the results presented here, while might be limited by the adopted model, can offer promising prospects for extrapolation to other cancer types possessing comparable settings. Moreover, the EAC model exhibits inherent heterogeneity and potential for metastasis, mirroring the clinical scenario observed in many cancer patients. This allows for the investigation of various aspects of tumor biology, including tumor heterogeneity, metastatic spread, and therapeutic responses, which may not be fully captured by the 4T1 model. Additionally, the fact that EAC solid tumor model can be employed in immunocompetent mice, provides an opportunity to explore the interactions between the tumor and the host immune system, unlike the xenograft models which while useful, do not fully capture the intricacies of the tumor microenvironment and immune responses that are critical to the progression and treatment response of TNBC. The xenograft approach also falls short in representing the genetic and cellular diversity of TNBC tumors due to the selection pressure imposed by the mouse host environment, which may lead to the overgrowth of certain cell types at the expense of others (Bleijs et al., 2019). So, we believe that by utilizing both the MDA-MB-231 cell line *in vitro* and the EAC solid tumor model *in vivo*, we were able to validate our *in vitro* findings in a more physiologically relevant context. This approach allows for the assessment of potential discrepancies between *in vitro* and *in vivo* responses and enhances the robustness and translatability of our findings in TNBC beside other potentially relevant contexts.

Furthermore, in accordance with the work by Mishra et al. (Mishra et al., 2018), the EAC model presents an unparalleled approach to scrutinize the deleterious impact of cancer chemotherapy on cardiac function. Leveraging the versatility of this model not only facilitates the elucidation of the pathophysiological mechanisms underpinning cancer-associated cardiac complications but also permits testing the adverse consequences of cancer chemotherapy in the heart. That being said, investigating the protective efficacy of CAR against DOX-induced cardiotoxicity within this model’s framework emerges as a compelling and viable avenue for exploration.

The current study has multiple limitations. One apparent caveat is related to the lack of behavioral studies that might have been utilized to validate successful stress induction. Also, another limitation is posed by the physiological systems activated in response to chronic restraint stress which not only include the sympathetic arm investigated herein, but also the hypothalamic-pituitary axis, with the stress hormone cortisol being the most amply studied in the context of stress-promoted cancer. In the present study, neither cortisol nor catecholamine levels were determined in blood or tumor, and as such, we cannot rule out the influence of corticosteroids on any of the estimated parameters. This is further compounded by the lack of any adrenoreceptor expression investigation. As alluded to earlier, most of the potential beneficial effects of CAR in the current study were likely attributed to β_2_-adrenergic blockade since this adrenoreceptor has been most explored in relevant studies. That being the case, CAR is a non-selective β and an α_1_-adrenoreceptor blocker. Since the inhibitory effects of the presynaptic α_2_ receptor are spared from CAR blockade, most of the endogenous (undetermined) catecholamines would hypothetically be directed towards this subtype. This notion is substantiated by experimental evidence showing that α_2_ blockade could fully recapitulate the impact of stress on breast cancer growth (Lamkin et al., 2015) suggesting another intriguing avenue for future investigation. Moreover, in order to validate that the favorable effects observed with CAR were due to its β-blockade potential, testing the effect of the S (−) enantiomer that inhibits β1, β2, and α1 adrenoceptors against the R (−) enantiomer that preferentially inhibits α1 adrenoceptor is recommended.

**FIGURE 8:**
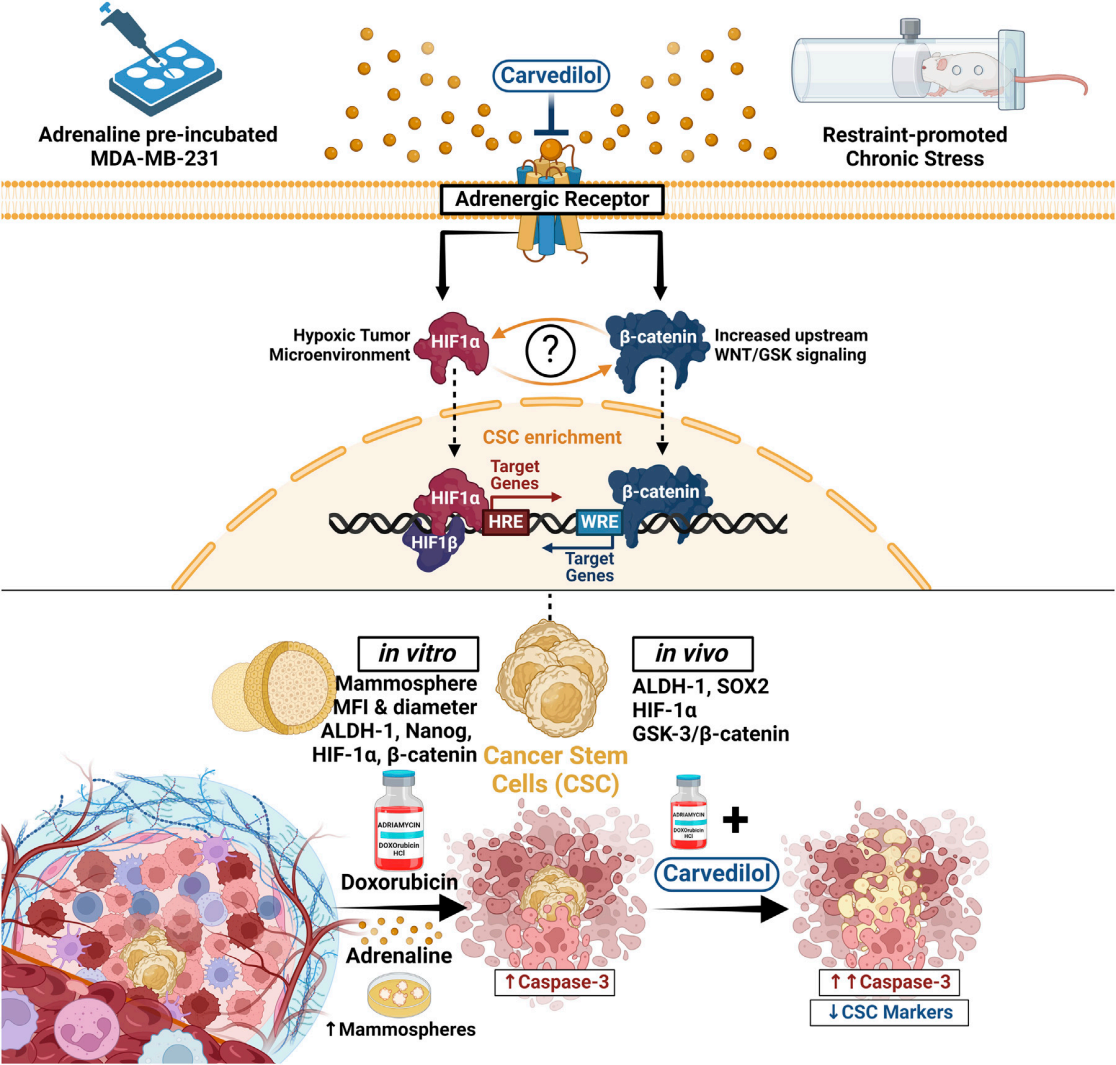
In this study, we delineated the sequence of events whereby adrenergic signaling influences DOX resistance and CSCs dynamics in TNBC. Utilizing both ADR-incubated MDA-MB-231 TNBC cells and a solid tumor model in mice under chronic stress, we observed that ADR treatment not only bolstered TNBC cell survival but also promoted CSC-like properties, as demonstrated by enhanced mammosphere formation and a surge in CSC markers, ALDH-1 and Nanog. The latter was coupled with an upregulation in HIF-1α and β-catenin levels. These effects were offset by combining the adrenergic blocker, CAR, to DOX. In stressed mice, tumors grew more rapidly and reached larger sizes, however, this effect was mitigated by CAR, which augmented DOX’s ability to suppress tumor growth. Central to our findings was the identification of HIF-1α as a pivotal mediator, connecting stress-induced sympathetic activation to the GSK-3β/β-catenin pathway, consequently promoting CSCs enrichment and DOX resistance, effects that were counteracted by CAR. Our research thus provides critical insights into CAR’s potential as an anticancer agent and highlights the synergy of CAR with DOX in treating TNBC. ALDH-1, Aldehyde dehydrogenase-1; CAR, Carvedilol; CSC, Cancer stem cells; DOX, Doxorubicin; GSK, Glycogen synthase kinase; HIF-1α, Hypoxia inducible factor 1 alpha; HRE, Hypoxia response elements; WRE, Wnt response elements.

In conclusion, we aimed to recapitulate the effects of adrenoreceptor blockade on DOX efficacy/chemoresistance *in vitro* using ADR-incubated MDA-MB-231 TNBC cells and *in vivo* utilizing a solid tumor mouse model of breast cancer promoted by chronic restraint stress. We showed that ADR treatment increased TNBC survival while simultaneously inducing a phenotypic switch reminiscent of CSCs, which was evident by their augmented capacity to efficiently form mammospheres. We further showed that tumors in stressed mice exhibited accelerated growth and attained greater sizes compared to their unstressed counterparts and that adrenergic blockade using CAR enhanced the impact DOX had on halting tumor growth. The current study also unveiled a center-stage role for HIF-1α linking stress-induced sympathetic activation to CSCs enrichment mediated through GSK-3β/β-catenin pathway undermining DOX resistance by CAR-induced adrenergic blockade. Our findings provide novel insights into the molecular mechanisms of CAR as an anticancer agent and open new vista for its clinical application in combination with DOX in TNBC. This study also lays the groundwork for more comprehensive studies that could include thorough adrenoreceptor profiling to further validate and extend these findings in stress-promoted cancer settings. Figure 8 provides a concise visual overview of our study’s methodology and key findings.

## Data Availability

The raw data supporting the conclusion of this article will be made available by the authors, without undue reservation.
